# Low expression of CXCR1/2 on neutrophils predicts poor survival in patients with hepatitis B virus-related acute-on-chronic liver failure

**DOI:** 10.1038/srep38714

**Published:** 2016-12-15

**Authors:** Ruonan Xu, Chunmei Bao, Huihuang Huang, Fang Lin, Yue Yuan, Siyu Wang, Lei Jin, Tao Yang, Ming Shi, Zheng Zhang, Fu-Sheng Wang

**Affiliations:** 1 Treatment and Research Centre for Infection Disease, Beijing 302 Hospital, Beijing 100039, China; 2 The Institute of Clinical Examination Centre, Beijing 302 Hospital, Beijing 100039, China; 3 The Institute of Intensive Care Unit, Beijing 302 Hospital, Beijing 100039, China

## Abstract

Polymorphonuclear neutrophils (PMNs) and proinflammatory cytokines have been implicated in the pathogenesis of acute-on-chronic liver failure (ACLF). But the utility of CXC chemokine receptor expression on PMNs as a biomarker for prediction of disease severity is still uncertain. In this study, we investigated the dynamic expression of CXCR1 and CXCR2 on neutrophils, and found that patients with hepatitis B virus-related ACLF displayed low expression of CXCR1 and CXCR2 on peripheral neutrophils compared with healthy subjects and patients with chronic hepatitis B. This expression pattern was correlated with disease severity. Additionally, increased production of IL-8 in peripheral blood was significantly associated with reduced CXCR1 and CXCR2 expression, as shown by the decreased CXCR1 and CXCR2 expression on neutrophils after treating neutrophils with plasma from ACLF patients. This effect could be overcomed through IL-8 blockage with an anti-IL-8 antibody. We also found that IL-8 production and neutrophil infiltration were coordinately increased in the liver tissue of HBV-ACLF patients, and this increase was associated with liver inflammation. Overall, increased production of IL-8 associated with neutrophils infiltration into the liver and decreased CXCR1/2 expression on peripheral neutrophils. CXCR1 and CXCR2 expression levels could be served as early markers to predict the severity of ACLF.

Acute-on-chronic liver failure (ACLF) is a devastating syndrome with high mortality, which encompasses an acute deterioration of liver function and is associated with the failure of other organs. Chronic hepatitis B virus (HBV) infection has been identified as the leading cause of ACLF in the Asia-Pacific region^1^. HBV-related ACLF (HBV-ACLF) is characterized by acute liver injury due to pre-existing chronic HBV (CHB) infection. Liver transplantation is the most effective therapy currently available, but very few patients can benefit from the transplantation due to a shortage of donors and high costs. Both early diagnosis and early prediction of outcomes are critical for improving survival.

Mounting evidence suggests neutrophil dysfunction displays a central role in liver injury^2^. Functional failure of neutrophils has been reported in a proportion of patients with cirrhosis and alcoholic hepatitis and these defects are associated with an increased risk of infection, organ failure, and mortality^3^. Neutrophils are recruited to the sites of inflammation through IL-8 and its two G protein–coupled chemokine receptors CXCR1 and CXCR2^4^. Neutrophils activated by IL-8 are considered as an important line of defense in the innate host immune response against bacterial infections^5^. Functionally significant changes in CXCR1 and CXCR2 have been described in infectious diseases and human melanoma^6^,^7^, but studies on the changes of CXCR1 and CXCR2 on neutrophils and the crosstalk between IL-8 and CXCR1/2 are still lacking in ACLF patients.

In order to investigate the dynamics of CXCR1 and CXCR2 expression on neutrophils in HBV-ACLF, we tested the production of IL-8, as well as CXCR1 and CXCR2 expression on neutrophils in our population of HBV-ACLF, CHB patients and healthy controls. We hypothesized that the expression of IL-8 would be elevated and the expression of CXCR1 and CXCR2 would be decreased in HBV-ACLF patients compared with CHB patients and healthy controls. Furthermore, in patients with poor outcomes (death or requiring liver transplantation), the decrease of CXCR1 and CXCR2 might be more significant compared with patients with good prognosis (survival). This study focused on the dynamics of CXCR1 and CXCR2 expression on neutrophils in HBV-ACLF patients and the crosstalk between IL-8 and CXCR1/2. Our results highlight the role of CXC chemokine receptors in predicting the outcomes of HBV-ACLF patients.

## Results

### Patient characteristics

Fifty-one patients were included in the HBV-ACLF cohort. The mean age was 42.86 ± 6.53. Our cohort included 44 men (86.2%) and 7 (13.7%) women. Of the total patients, 64.7% (n = 33) were cirrhotic and 35.2% (n = 18) were non-cirrhotic. The results of a 90 day follow-up study showed that 28 HBV-ACLF patients survived and 23 patients died, giving a survival rate of 54.9%. Significant differences were found between survivors and non-survivors in terms of total bilirubin, INR (international normalized ratio), Model for end-stage liver disease (MELD) and Sequential organ failure assessment (SOFA) scores. As shown in Table 1, HBV-ACLF patients had significantly higher level of serum liver enzymes, serum total bilirubin (TBil), INR, MELD scores and SOFA scores.

### CXCR1 and CXCR2 expression on neutrophils are decreased in HBV-ACLF patients

**Figure 1:**
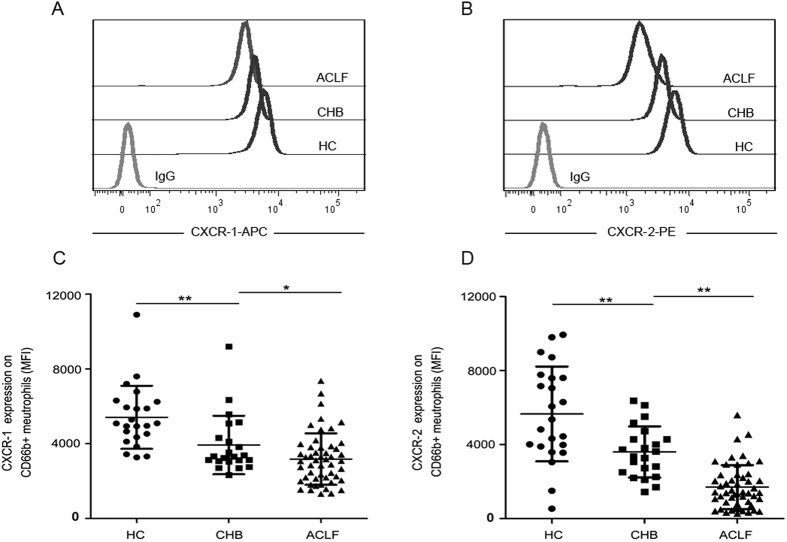
Expression of CXCR1 and CXCR2 on neutrophils from ACLF patients and controls. (**A**) Representative dot plots show the expression of CXCR1 on CD66b-positive cells in patients and healthy controls. (**B**) Representative dot plots show the expression of CXCR2 on CD66b-positive cells in patients and healthy controls. (**C**) Pooled data showing the MFI of CXCR1 expression on CD66b-positive cells in HC, CHB and HBV-ACLF patients. (**D**) Pooled data showing the MFI of CXCR2 expression on CD66b-positive cells in HC, CHB and HBV-ACLF patients. Each circle represents an individual. *P < 0.05; **P < 0.01.

We first measured CXCR1 and CXCR2 expression levels on peripheral neutrophils in all subjects. As shown in Fig. 1A and B, the mean fluorescence intensity (MFI) of CXCR1 and CXCR2 expression on neutrophils was lower in HBV-ACLF patients when compared with CHB patients, and significantly decreased as compared with healthy controls (Fig. 1C and D). In order to adjust for the effects of cirrhotic status, we further analyzed CXCR1 and CXCR2 expression levels in HBV-ACLF patients by stratification based on cirrhosis status; however, no significant differences were found (Supplementary Figure 1).

### CXCR1 and CXCR2 expression levels are significantly correlated with SOFA and MELD scores

**Figure 2:**
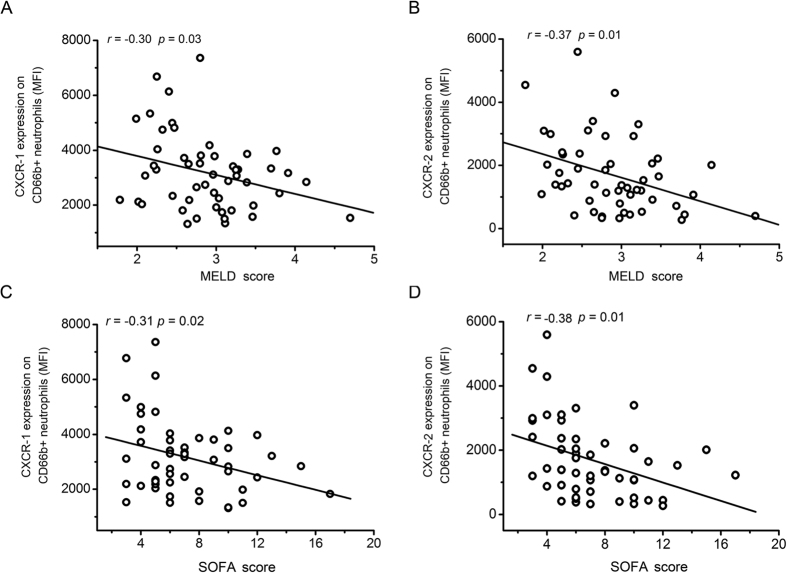
Reductions in CXCR1 and CXCR2 expression are negatively associated with the severity of ACLF. (**A**) CXCR1 levels are negatively correlated with the MELD score of ACLF patients. (**B**) CXCR2 levels are negatively correlated with the MELD score of ACLF patients. (**C**) CXCR1 levels are negatively correlated with the SOFA score of ACLF patients. (**D**) CXCR2 levels are negatively correlated with the SOFA score of ACLF patients. Each circle represents an individual.

To analyze the clinical significance of CXCR1 and CXCR2 expression with the well-established liver injury markers and organ failure markers, we investigated whether there was a correlation between lower CXCR1 and CXCR2 expression levels in HBV-ACLF patients with MELD and SOFA scores. As shown in Fig. 2A and B, CXCR1 (r = −0.30, 95% confidence interval is −0.53 to −0.03) and CXCR2 (r = −0.37, 95% confidence interval is −0.59 to −0.11) expression was negatively correlated with the MELD score, the expression of CXCR1 (r = −0.31, 95% confidence interval is −0.54 to −0.04) and CXCR2 (r = −0.38, 95% confidence interval is −0.59 to −0.12) was also negatively correlated with the SOFA score (Fig. 2C and D). We further analyzed the hazards for the death of patients with HBV-ACLF by Cox’s regression analysis (Table 2). Our data showed that the serum TBil, INR, MELD, SOFA and CXCR1 MFI were the independent risk factors for the death of HBV-ACLF patients.

### CXCR1 and CXCR2 expression levels are correlated with the survival of HBV-ACLF patients

**Figure 3:**
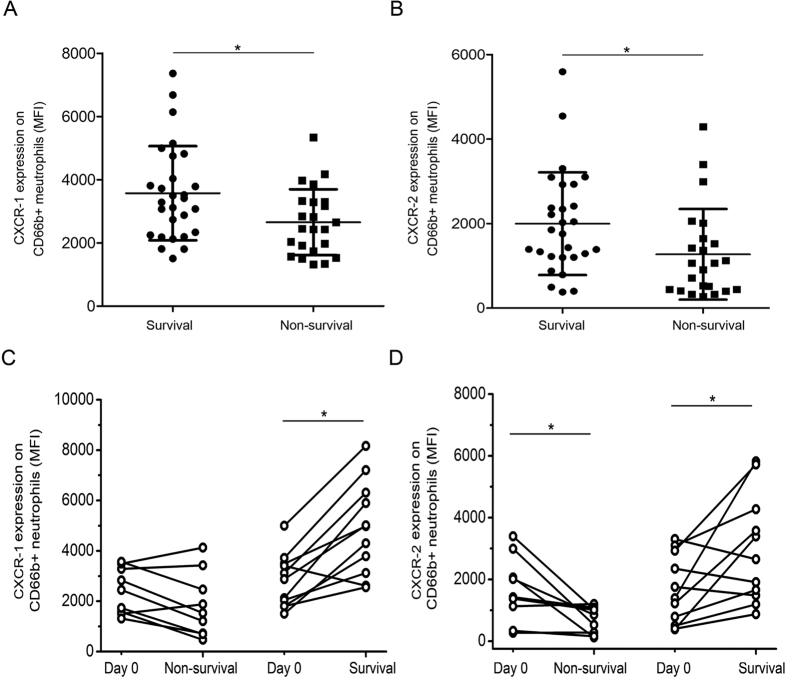
CXCR1 and CXCR2 levels are prognostic markers for survival in ACLF patients. (**A**) CXCR1 levels are associated with mortality in ACLF patients. (**B**) CXCR2 levels are associated with mortality in ACLF patients. (**C**) An increase in CXCR1 expression on CD66b-positive cells is correlated with improvement in ACLF patients. (**D**) An increase in CXCR2 expression on CD66b-positive cells is correlated with improvement in ACLF patients. Each circle represents an individual. *P < 0.05; **P < 0.01.

Based on our observations of decreased CXCR1 and CXCR2 expression levels in HBV-ACLF patients, we performed a 90 day follow-up to establish the relationship between expression of the markers and survival outcomes. As shown in Fig. 3A and B, CXCR1 and CXCR2 expression levels on neutrophils at admission could stratify patients into survival and non-survival groups. In the follow-up study, HBV-ACLF patients (n = 11) showed significant increases in both CXCR1 and CXCR2 expression as they recovered from the disease (Fig. 3C and D). In contrast, there was an obvious decline in CXCR2 expression when patients (n = 9) did not recover from ACLF.

### HBV-ACLF patient plasma reduces CXCR1 and CXCR2 expression on neutrophils from healthy donors

**Figure 4:**
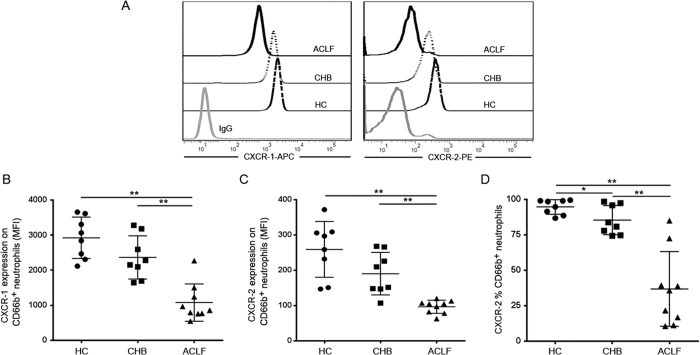
Effects of ACLF patient plasma on CXCR1 and CXCR2 expression on neutrophils from healthy donors. (**A**) Representative dot plots show the MFI of CXCR1 and CXCR2 expression on CD66b-positive cells in normal neutrophils incubated with ACLF patient plasma. (**B**) Pooled data show the MFI of CXCR1 expression on CD66b-positive cells when exposed to ACLF patient plasma *in vitro*. (**C**) Pooled data show the MFI of CXCR2 expression on CD66b-positive cells when exposed to ACLF patient plasma *in vitro*. (**D**) Pooled data show the percentage of CXCR-2 expression on CD66b-positive cells when exposed to ACLF patient plasma *in vitro*. Data indicate the means and standard deviations. *P < 0.05; **P < 0.01.

To identify the reason for the decrease of CXCR1 and CXCR2 expression on peripheral neutrophils in HBV-ACLF patients, we incubated neutrophils from healthy patients with plasma from HBV-ACLF patients. We found that the healthy neutrophils showed decreased CXCR1 and CXCR2 expression when co-cultured with plasma from ACLF patients (Fig. 4A). The difference between ACLF patients and CHB patients was most notable (Fig. 4B and C). In addition to the MFI of CXCR2, the percentage of CXCR2 expression was also significantly reduced (Fig. 4D). These results indicate that the phenotype of neutrophils can be altered by plasma transfer.

### Increased IL-8 production is associated with CXCR1 and CXCR2 expression decrease in HBV-ACLF patients

**Figure 5:**
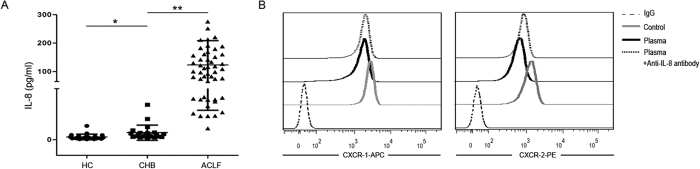
Increased IL-8 production is associated with decreased CXCR1 and CXCR2 expression on neutrophils. (**A**) Pooled data show the plasma levels of IL-8 in HC, CHB and HBV-ACLF patients. (**B**) Representative dot plots show the expression of CXCR1 and CXCR2 on normal neutrophils incubated with ACLF patient plasma and after the ACLF patient plasma was blocked by an anti-IL-8 antibody. *P < 0.05; **P < 0.01.

To further explore the mechanism whereby the expression of CXCR1 and CXCR2 on neutrophils is reduced, we analyzed IL-8 production in the plasma. As shown in Fig. 5A, IL-8 levels were much higher in HBV-ACLF patients than in CHB patients and healthy controls. Most importantly, the reduction in CXCR1 and CXCR2 expression induced by the addition of HBV-ACLF patient plasma could be blocked by anti-IL-8 antibody (Fig. 5B). This suggests that increased IL-8 is the key factor mediating the decreased expression of CXCR1 and CXCR2.

### IL-8 positive cells and neutrophils accumulate in the livers of HBV-ACLF patients

**Figure 6:**
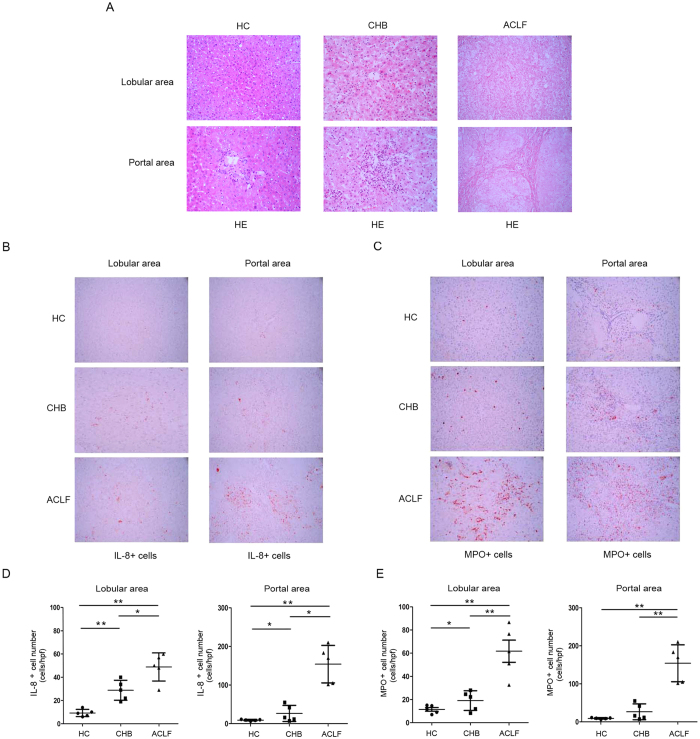
*In situ* liver infiltration of IL-8-producing cells and neutrophils are associated with liver injury in ACLF patients. (**A**) HE staining of liver tissues from HC control, CHB and ACLF patients. (**B**) *In situ* immunohistochemical staining for IL-8 in patients with various degrees of liver injury (400X). (**C**) Immunohistochemical staining of MPO–positive cells in liver samples from patients (400X). (**D**) Numbers of IL-8–positive cells in liver portal and lobular areas are shown in patients with various liver injury. (**E**) Numbers of MPO–positive cells in liver portal and lobular areas are shown. Each dot represents one individual. *P < 0.05; **P < 0.01.

Beyond detecting changes in IL-8 and neutrophils in the peripheral blood, we also examined the distribution of IL-8 cells and neutrophils in the liver. As shown by HE staining (Fig. 6A), inflammatory cells infiltration and hepatocytes necrosis were obvious in the liver of ACLF patients. In addition, an increased number of IL-8 cells were found in the liver of ACLF patients, whereas the liver tissue from healthy donors and CHB patients had few IL-8-secreting cells (Fig. 6B and D). With the increase of IL-8 production, neutrophils infiltration was also clearly observed when compared with HC and CHB patients (Fig. 6C and E), which was identified by myeloperoxidase staining. Our data suggest that large amounts of neutrophils infiltration into liver induced by IL-8 over production might eventually cause severe liver injury.

## Discussion

Neutrophils are the first line of defense against environmental challenges and injury. Impaired peripheral immune responses to microbial challenges, termed immunoparesis, is postulated to be responsible for the development of secondary infections, and is an independent predictor of mortality in patients with ACLF^8^. Neutrophil dysfunction is an important biomarker in predicting the outcome of alcoholic hepatitis superimposed on cirrhosis^9^. In clinic, using granulocyte-colony stimulating factor therapy to mobilize CD34+ cells and restore neutrophil function provides potential benefits for patients with ACLF^10^,^11^. Maintenance of neutrophil function is clearly important and the identification of novel neutrophil markers is urgently needed to provide prompt prognosis to increase survival of HBV-ACLF patients.

In clinical assessments of the outcomes of patients with cirrhosis during an episode of ascites, the presence of bacterial DNA and a MELD score lower than 15 were indicators of ACLF and did not predict the development of spontaneous bacterial peritonitis^12^. These phenomena might highlight that clinical deterioration in patients is more important. Except for MELD score other immunological markers for the early prediction of outcomes in ACLF patients are not routinely utilized. Two CXC chemokine receptors, CXCR1 and CXCR2, have been reported to mediate the response to CXC chemokines in PMNs, and IL-8 was regarded as the prototypic CXC chemokine mediating chemotaxis, transendothelial migration and the activation of neutrophils^13^. This study focused on the dynamics of CXCR1 and CXCR2 expression in ACLF patients and the crosstalk between IL-8 and CXCR1/2 expression on neutrophils. Our data provided four pieces of evidence to support an association between reductions in CXCR1 and CXCR2 expression and ACLF progression. First, the expression levels of CXCR1 and CXCR2 were dramatically reduced in HBV-ACLF patients when compared with CHB patients and HC subjects. Second, there was a significant negative correlation between the expression of CXCR1 and CXCR2 on neutrophils with MELD and SOFA scores. Third, low levels of CXCR1 and CXCR2 expression on neutrophils were associated with high mortality of ACLF patients. Fourth, the expression levels of CXCR1 and CXCR2 were significantly increased as ACLF patients improved in a long-term follow-up. These data suggest that the expression of CXCR1 and CXCR2 on neutrophils may serve as prognostic markers for predicting the outcomes of HBV-ACLF patients. Except for the correlation analysis, we also analyzed hazards for the death of patients with ACLF by Cox’s regression analysis, TBIL, INR and MELD scores as independent risk factors for the death of ACLF patients were demonstrated in many studies, here our studies found that CXCR1 MFI was also an independent risk factor for the death of ACLF, but MFI of CXCR2 did not show significant difference, the reason might due to the CXCR2 has the same ligand with CXCR1.

The mechanisms underlying CXCR1 and CXCR2 downregulation remain to be investigated. Previous experiments have suggested that binding of IL-8 could lead to reduced surface expression of CXCR1, rendering cells resistant to subsequent challenge with IL-8^14^. Here, we showed that decreased CXCR1 and CXCR2 expression could be induced in healthy neutrophils with the addition of plasma from HBV-ACLF patients, while an anti-IL-8 antibody could block these effects. The results are supported by the increased levels of IL-8 production in the plasma of HBV-ACLF patients, which might account for decreased CXCR1 and CXCR2 expression through rapid receptor internalization. In addition to IL-8 production, we also found that lipopolysaccharide (LPS) was significantly increased in the plasma of HBV-ACLF patients (Supplymentary Fig. 2A). *In vitro*, LPS can decrease CXCR1 and CXCR2 expression (Supplymentary Fig. 2B); however, an anti-CD14 antibody failed to restore CXC receptor expression when neutrophils were cultured with patient plasma (Supplymentary Fig. 2C). These findings suggest that, in HBV-ACLF patients, downregulation of CXCR1 and CXCR2 expression on neutrophils might be IL-8 but not LPS dependent.

Liver inflammation, hepatocyte necrosis are key pathologies in liver failure. In the acetaminophen (APAP) liver model, neutrophils are recruited into the liver closely following the development of cell injury 4–24 h after drug treatment. This triggers complete neutrophil activation with prolonged adherence-dependent oxidative stress and degranulation^15^, through direct killing of hepatocytes or through the expression of FasL inducing hepatocytes apoptosis^16^. Blockage of neutrophil infiltration by combined CXCR2-FPR1 antagonism prevents hepatotoxicity^17^. To further examine neutrophil dynamics in ACLF patients, we analyzed IL-8 production, and CXCR1 and CXCR2 expression on neutrophils in liver samples *in situ*. We observed that increased neutrophil infiltration and IL-8 production synchronized in ACLF patients.

CXCR1 and CXCR2 have different ligand binding affinities, CXCR1 binding with high affinity to IL-8, and CXCR2 binding all CXC chemokines^18^. In our study, we found that CXCR1 and CXCR2 expressed in the peripheral blood were associated with the outcome of HBV-ACLF patients, while for Cox’s regression analysis, CXCR1 has significancy, which meant that CXCR1 might be more important than CXCR2. Given the crucial role of neutrophils in ACLF, we investigated additional receptors including TLR-2, 4, phagocytic capacity and the oxidative burst activity of neutrophils. Decreased TLR-4 was also identified (Supplymentary Fig. 3). These results highlight neutrophil dysfunction in ACLF and indicate that evaluating neutrophil function is important for predicting outcomes of ACLF patients.

In summary, we systematically examined changes in CXCR1 and CXCR2 expression on neutrophils in ACLF patients in a cross-sectional and follow-up study. Our results demonstrate that elevated IL-8 mediates downregulation of CXCR1/2 expression on neutrophils and is involved in neutrophil-mediated liver injury. Those findings identify the autocrine loop of IL-8 and CXCR1/2 in ACLF patients, and highlight the role of CXCR1 and CXCR2 in predicting the outcome of HBV patients with ACLF.

## Methods

### Study subjects

Seventy-three patients including 51 HBV-ACLF patients, 22 CHB patients and 23 healthy controls were enrolled from March 2013 to October 2015 at the Beijing 302 hospital. Chronic hepatitis B patients were diagnosed based on the criteria of the Viral Hepatitis Management Scheme, issued by the Chinese Society of Infectious Diseases and the Chinese Medical Association of Hepatology. Individuals with concurrent hepatitis C virus, hepatitis G virus, or HIV-1 infection were excluded. Diagnosis for HBV-ACLF patients was based on consensus recommendations of the Asia-Pacific Association for The Study of the Liver (APASL). Inclusion criteria were: (1) Clear acute hepatic insult manifesting as jaundice and coagulopathy, complicated within 4 weeks by ascites and/or encephalopathy, in a patient with previously diagnosed or undiagnosed chronic liver disease. (2) Total bilirubin ≥5 mg/dL (85 μmol/L). (3) Prothrombin activity ≤40% or INR ≥1.5. (4) The presence of ascites and/or encephalopathy as determined by physical examination. The APASL definition was chosen because it is more suitable for Asian patients, particularly for Chinese ACLF patients, those were mainly CHB patients. All HBV-ACLF patients were followed up for 90 days to identify the clinical outcome, and patients were groups into survival and non-survival. The study protocol was approved by the ethics committee of 302 Hospital which complied with the declaration of Helsinki, and written informed consent was obtained from each subject.

### Blood samples and laboratory data collection

Blood samples were collected within 12 h of the HBV-ACLF diagnosis at our hospital. The following data were recorded for each patient: age, sex, HBeAg status; HBV DNA (IU/mL); cirrhosis; total bilirubin (TB; μmol/L); prothrombin activity; alanine aminotransferase (ALT; IU/L); albumin (g/L); blood platelet count (BPC); creatinine (μmol/L); model for the end-stage liver disease score and organ failure assessment (SOFA) score. Survival or death was assessed during a follow-up period of 90 days. Blood was obtained at day 0, and every two weeks thereafter, and when the patient left the hospital (death or survival). Blood samples from 22 CHB patients and 23 healthy controls were collected at enrollment. Blood plasma was separated and stored at −80 °C after collection.

### Flow cytometry and ELISA assays

Fluorescein-conjugated antibodies were provided by Biolegend (Biolegend, San Diego CA, USA). Fresh heparinized peripheral blood (100 μL) was incubated with CXCR-1 and CXCR-2 antibodies or corresponding isotype controls according to the manufacturer’s instructions. CD66b positive identified granulocytes in the whole blood. The specificity of the CXCR1 and CXCR2 antibodies were already described at elsewhere^19^. The cells were analyzed using a calibur flow cytometer and CELLQuest software as previously mentioned^20^. Plasma collected from patients and healthy controls was used to measure the circulating concentration of IL-8 (eBioscience, San Diego CA, USA) according to the manufacturer’s instructions.

### Neutrophil isolation and incubation with plasma

Neutrophils were harvested from healthy volunteers and purified by Ficoll-Paque gradient centrifugation and dextran sedimentation. After hypotonic lysis of residual red blood cells, the neutrophils were used for further studies. Neutrophils were counted and resuspended in 1640 medium at a density of 5 × 10^5^ in 50 μL. Fifty microliters of cell suspension and 50 μL of plasma were used in each assay. Following an incubation at 37 °C for 2 h, CXCR-1 and CXCR-2 expression on CD66b positive cells were examined.

For anti-IL-8 antibody blocking, plasma from ACLF patient was incubated with anti-IL-8 antibody (Abcam, Camdridge, UK) at a concentration of 0.1 μg/mL for 1 h at room temperature, and then incubated with neutrophils for an additional 2 h to evaluate CXCR-1 and CXCR-2 expression.

### Immunohistochemical staining

Paraffin-embedded, formalin-fixed liver tissue (≥5 μm) was firstly stained by hematoxylin and eosin, and then these tissues were incubated with anti IL-8 antibody (Abcam, Cambridge, UK) overnight at 4 °C after blocking endogenous peroxidase activity with 0.3% H_2_O_2_. Myeloperoxidase (Jingmei Biotech, BeiJing, China) was used to stain neutrophils. Positively stained cells were counted using a high-power microscope (400X magnification) according to standard protocols^21^.

### Statistical analysis

Data analysis was performed with SPSS version 13.0 software (SPAA Inc., Chicago, IL, USA) and data were expressed as means ± standard deviation. Statistically significant differences between two groups were determined by the Mann–Whitney nonparametric U-test. Comparisons of data from the same individual were performed using the Wilcoxon matched-pairs T test. Correlation analyses were determined by the Spearman rank correlation test. Multivariate Cox regression was performed to identify independent risk factors for poor prognosis of HBV-ACLF. Values of P < 0.05 were considered as statistically significant.

## Additional Information

**How to cite this article**: Xu, R. *et al*. Low expression of CXCR1/2 on neutrophils predicts poor survival in patients with hepatitis B virus-related acute-on-chronic liver failure. *Sci. Rep.* **6**, 38714; doi: 10.1038/srep38714 (2016).

**Publisher's note:** Springer Nature remains neutral with regard to jurisdictional claims in published maps and institutional affiliations.

## Supplementary Material

Supplementary Information

## Figures and Tables

**Table 1:** Characteristics of study subjects.

	HC (n = 23)	CHB (n = 22)	ACLF (n = 51)			
			Total (n = 51)	Survival (n = 28)	Non-survival (n = 23)	P
Age (years)	34.15 ± 4.13	45.35 ± 4.51	42.86 ± 6.53	40.32 ± 4.51	46.12 ± 4.22	NS
Male (%)	12 (52.1%)	17 (77.2%)	44 (86.2%)	24 (85.7%)	20 (86.9%)	NS
HBeAg positive n (%)	/	12 (54.5%)	24 (47.1%)	14 (58.3%)	10 (41.7%)	NS
Log_10_HBV DNA (IU/mL)	/	3.78 ± 0.51	4.33 ± 0.91	4.07 ± 0.33	4.97 ± 1.01	NS
Cirrhotic n (%)	/	/	33 (64.7%)	19 (67.8%)	14 (60.8%)	NS
TB (*μ*mol/L)	10.12 ± 0.21	36.67 ± 3.41	380.67 ± 32.76	340.23 ± 22.45	440.19 ± 39.08	<0.05
INR	/	0.99 ± 0.07	2.01 ± 0.41	1.89 ± 0.31	2.39 ± 0.67	<0.05
ALT (IU/L)	21.37 ± 4.45	114.27 ± 22.45	386.13 ± 39.24	296.13 ± 37.25	500.13 ± 46.65	<0.05
Albumin (g/L)	45.37 ± 2.75	39.67 ± 6.55	26.69 ± 3.87	29.33 ± 4.67	24.69 ± 5.82	NS
BPC (×10^9^)	/	201.87 ± 22.75	99.17 ± 9.75	113.47 ± 20.15	83.42 ± 25.65	NS
Cre (mg/dL)	/	0.97 ± 0.13	1.25 ± 0.21	1.34 ± 0.19	1.22 ± 0.31	NS
MELD score	/	/	2.88 ± 1.19	2.72 ± 1.01	3.21 ± 1.47	<0.05
SOFA score	/	/	7.18 ± 2.99	5.61 ± 1.72	9.00 ± 2.98	<0.05

Data are presented as values (percentage) or mean ± SD. Abbreviations: HC, healthy controls; CHB, chronic hepatitis B; ACLF, acute-on-chronic liver failure; HBeAg, hepatitis Be antigen; TB, total bilirubin; INR, international normalized ratio; BPC, blood platelet counts; Cre, Creatinine; MELD score, model for the end-stage liver disease score; SOFA score, sepsis-related organ failure assessment score; NS, not significant. *P values correspond to the comparison of the Survival and Non-survival groups of HBV-ACLF patients.

**Table 2:** Cox’s regression analysis of the HBV-ACLF cohorts.

	P	HR	95.0% CI
ALT	0.108	1.003	0.999–1.007
AST	0.053	0.994	0.988–1.000
TBIL	0.046	1.009	1.000–1.019
INR	0.010	21.846	2.095–227.839
CXCR1	0.033	1.001	1.000–1.002
CXCR2	0.511	1.000	0.999–1.001
MELD	0.027	0.054	0.004–0.720
SOFA	0.047	1.311	0.991–1.735

HR, hazard ratio; CI, confidence interval; ALT, alanine aminotransferase; AST, glutamic-pyruvictransaminase.
