# Supplementary material for: Low expression of CXCR1/2 on neutrophils predicts poor survival in patients with hepatitis B virus-related acute-on-chronic liver failure
Source: Sci Rep. 2016 Dec 15;6:38714. doi: 10.1038/srep38714 (PMC5156931; doi:10.1038/srep38714)
Supplement: Supplementary Information [file srep38714-s1.doc]

Low expression of CXCR1/2 on neutrophils predicts poor survival in patients with hepatitis B virus-related acute-on-chronic liver failure

Ruonan Xu*^1^，Chunmei Bao *^2^, Huihuang Huang *^1^, Fang Lin^3^, Yue Yuan^1^, Siyu Wang^1^, Lei Jin^1^, Tao Yang^1^, Ming Shi^1^, Zheng Zhang^1^, Fu-Sheng Wang^1#^

1 Treatment and Research Centre for Infection Disease, Beijing 302 Hospital, Beijing 100039, China. 2 The Institute of Clinical Examination Centre, Beijing 302 Hospital, Beijing 100039, China. 3 The Institute of Intensive Care Unit, Beijing 302 Hospital, Beijing 100039, China.

*These authors contributed equally to this study.

^#^Corresponding author: Fu-Sheng Wang. fswang302@163.com. Treatment and Research Centre for Infection Disease, Beijing 302 Hospital, Beijing 100039, China.


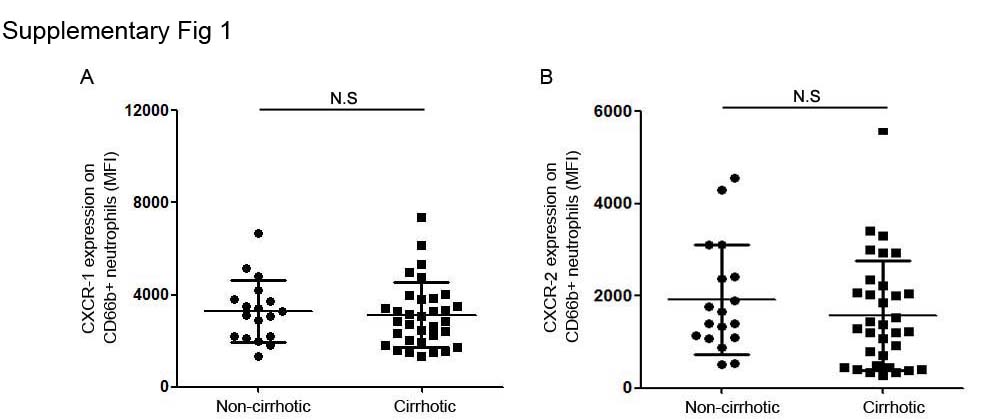


Expression of CXCR1 and CXCR2 on neutrophils from ACLF patients with cirrhosis and without cirrhosis. (A) Pooled data show the MFI of CXCR1 expression on CD66b-positive cells in HBV-ACLF patients with cirrhosis and without cirrhosis. (B) Pooled data show the MFI of CXCR2 expression on CD66b-positive cells in HBV-ACLF patients with cirrhosis and without cirrhosis. Each circle represents an individual. N.S represents no significance.


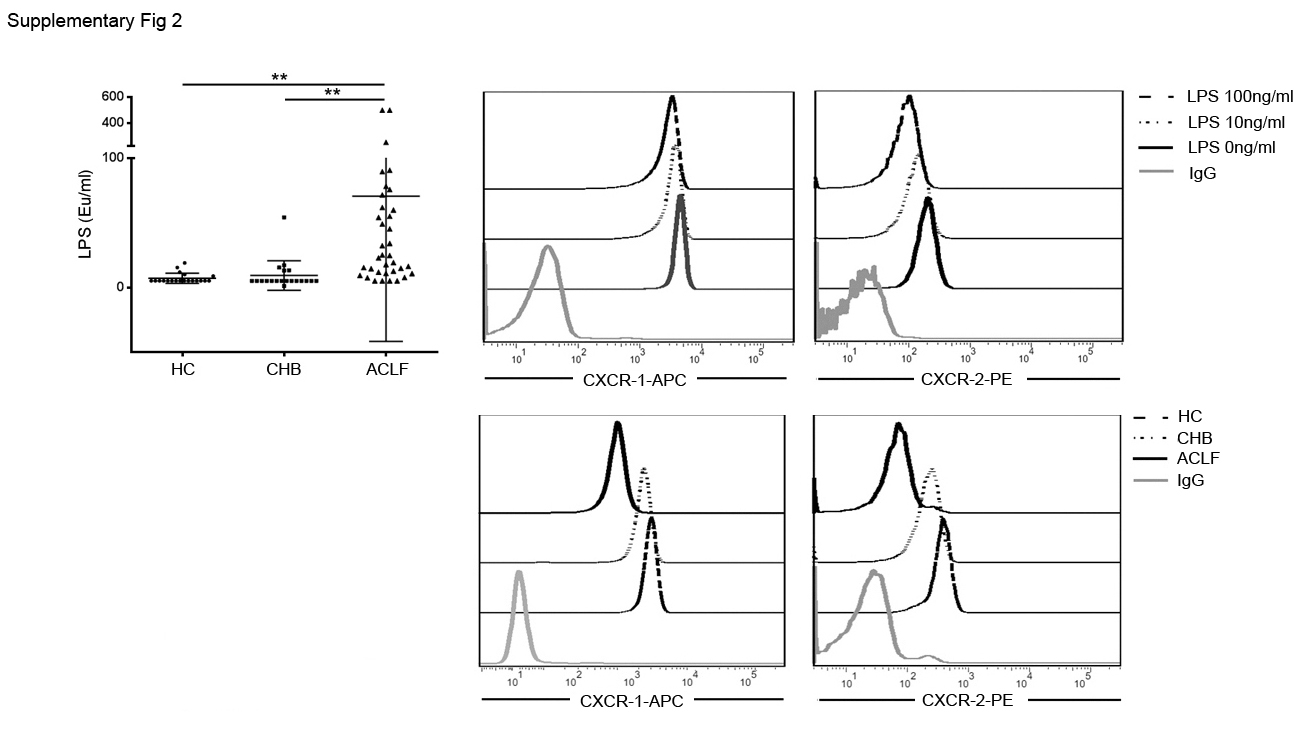


Increased LPS production is not associated with decreased CXCR1 and CXCR2 expression on neutrophils. (A) Plasma levels of LPS are increased in ACLF patients. (B) LPS has the ability to reduce CXCR1 and CXCR2 expression on neutrophils from healthy donors. (C) Decreased CXCR1 and CXCR2 expression on normal neutrophils incubated with ACLF patient plasma can’t be reversed by anti-CD14 antibody blocking. * P < 0.05; ** P <0.01.


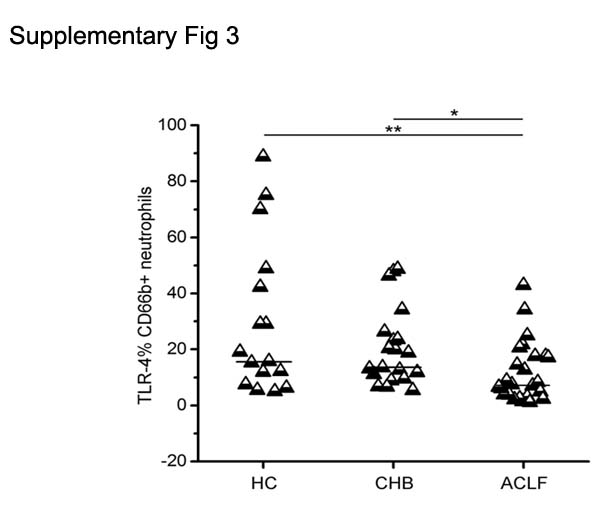


Decreased TLR-4 expression on CD66b-positive cells in HBV-ACLF patients. * P < 0.05; ** P <0.01.
